# Surface roughness and microhardness of 3D printed denture base resins when printed with different printers

**DOI:** 10.2340/biid.v13.45751

**Published:** 2026-05-05

**Authors:** Faisal D. Al-Qarni

**Affiliations:** Department of Substitutive Dental Sciences, College of Dentistry, Imam Abdulrahman Bin Faisal University, Dammam, Saudi Arabia

**Keywords:** 3D printing, additive manufacturing, denture base, digital light processing

## Abstract

**Objective:**

This study aimed to investigate the influence of using different printers on surface roughness and hardness of different 3D printed dentures.

**Methods:**

Three denture base resins (ASIGA, NextDent, and FormLabs) were printed with one of two printers: ASIGA or NextDent. Surface roughness was measured with a non-contact profilometer, while a surface hardness tester was used to measure Vicker’s hardness values. One-way and Two-way analysis of variance (ANOVA), and *t*-test were used to compare means with a significance level set at 0.05.

**Results:**

FormLabs and NextDent specimens showed different hardness values when different printers were used (*p* < 0.001), while ASIGA specimens had no difference (*p* = 0.072). Roughness values were similar with NextDent specimens printed with different printers (*p* = 0.053), while ASIGA and FormLabs specimens’ roughness values were significantly influenced by the printer used (*p* < 0.001). When printed with ASIGA, ASIGA and NextDent specimens had similar hardness values, which were higher than FormLabs specimens. When NextDent printer was used, ASIGA specimens showed superior hardness values compared to NextDent and FormLabs. When comparing surface roughness of different resin specimens printed with the same printer, differences were observed among all specimens (*p* < 0.001).

**Conclusions:**

The type and brand of the printer used can affect surface properties of printed dentures. When the same printer was used, surface roughness and hardness were significantly different among all resin materials. The choice of resin material and printer combination affects both surface roughness and microhardness.

**Clinical Significance:**

The choice of resin material and printer type may significantly impact the microhardness and surface roughness of 3D printed dentures. Careful material and printer selection is essential to ensure optimal clinical performance and longevity of prostheses.

## Introduction

Fabrication of removable dentures using digital technologies is increasingly popular [1, 2]. Due to continuous advances, digital dentistry has revolutionised the fabrication of dental prostheses in fixed, removable, and implant prosthodontics [3–6]. In complete dentures, digital workflows have introduced novel technical and clinical protocols with early interest in subtractive milling [7–10]. 3D printing is gaining more popularity because of lower initial cost, high cost-effectiveness, and the ability to fabricate more complex structures compared to subtractive milling [11–13].

Several 3D printing technologies have been utilised in the dental field [14–16]. Stereolithography (SLA) is one of the early introduced technologies, in which an ultraviolet (UV) light laser beam cures the liquid resin layer by layer [17]. Digital Light Processing (DLP) technology uses several micromirrors, which help cure the entire layer by a single laser irradiation, thus resulting in shorter printing time compared to SLA. Material jetting (MJ) involves spraying liquid droplets on the build platform, which is then cured with UV light. Other technologies include Selective Laser Sintering (SLS) that uses a laser to selectively fuse powder material layer by layer into a solid structure, and Fusion Deposition Modeling (FDM) in which the material is heated, melted, and extruded then solidified layer by layer [14–17].

The use of different printers has been reported to influence printing accuracy, as well as flexural strength [18–24]. A systematic review [25] assessed the accuracy of printed models and reported SLA, DLP, and MJ to be the most accurate 3D printing technologies. Another study [26] evaluated the accuracy of (12) printers and concluded that all printers produced acceptable accuracy. Srinivasan [27] evaluated resins printed with a manufacturer-recommended printer compared to a third-party printer and reported that the manufacturer-recommended printer had superior mechanical behaviour compared to the third-party printer. However, another study reported that resins designed for DLP produced superior results when printed with an SLA printer [28].

The influence of resin materials printed with different printers on surface properties remains unclear. Therefore, the purpose of this study was to assess microhardness and surface roughness of 3D printed denture base resins when printed with different printers. The null hypothesis states that the choice of resin material or printing technology has no significant influence on the hardness or surface roughness of 3D-printed denture base resins.

## Methods and materials

Based on previous similar studies [27, 29], a total of 120 specimens (*n* = 20) were deemed sufficient to achieve a satisfactory level of power. Disc specimens, measuring 10 × 2.5 mm, were digitally designed and converted into standard tessellation language (STL) files for 3D printing. Three resin materials were used in this study: ASIGA (DentaBASE, ASIGA, Erfurt, Germany), NextDent (Denture 3D+, NextDent B.V., Soesterberg, The Netherlands), and FormLabs (Denture Base Resin LP, Formlabs Inc, Somerville, MA, USA). Each material was printed with two different 3D printing systems: ASIGA MAX™ (ASIGA, Erfurt, Germany) and NextDent 5100 (NextDent B.V., Soesterberg, The Netherlands) . Detailed information regarding the study design, printing parameters, and specifications can be found in Tables 1 and 2.

**Table 1:** Denture base resin materials used in the study.

Material	Manufacturer	Composition
DentaBASE	ASIGA, Erfurt, Germany	- Ethoxylated bisphenol A dimethacrylate - 7,7,9(or 7,9,9)-trimethyl-4,13-dioxo-3,14-dioxa-5,12 diazahexadecane-1,16-diyl bismethacrylate - 2-hydroxyethyl methacrylate - Silicon dioxide - Diphenyl(2,4,6trimethylbenzoyl)-phosphine oxide - Titanium oxide
Denture 3D+	NextDent B.V., Soesterberg, The Netherlands	- Ethoxylated bisphenol A dimethacrylate - 7,7,9(or 7,9,9)-trimethyl-4,13-dioxo-3,14-dioxa-5,12 diazahexadecane-1,16-diyl bismethacrylate - 2-hydroxyethyl methacrylate - Silicon dioxide - Diphenyl(2,4,6trimethylbenzoyl)-phosphine oxide - Titanium oxide
Denture Base Resin LP	Formlabs Inc, Somerville, MA, USA	- 55–75% w/w urethane dimethacrylate - 15–25% w/w methacrylate monomers - 0.9% w/w phenyl bis (2,4,6-trimethylbenzoyl)-phosphine oxide

**Table 2:** 3D printers used.

3D Printer name	Printer type	Printing parameters
NextDent 5100	Figure 4 DLP	Printing orientations: 0-degree Printing layer thickness: 50 µm Post curing machine: LC-3DPrint Box Post curing time/Temp: 15 min/80°C
ASIGA MAX™	LED-based DLP	Printing orientations: 0-degree Printing layer thickness: 50 µm Post curing machine: Asiga Flash Post curing time/Temp: 20 m/60°C

Following the completion of the printing process, the support structures were eliminated, and the surface of the specimens was polished using silicon carbide paper with grit sizes of 800, 1,500, and 2,000. All specimens were immersed in distilled water for 48 (±2) h at 37°C before testing.

To assess the surface roughness of the specimens, a non-contact profilometer (Contour Gt-K1 optical profiler; Bruker Nano, Inc., Tucson, AZ, USA) was employed. The profilometer scanned five different points on each specimen, and the average surface roughness (µm) per specimen was then calculated.

For the hardness test, a Vickers tester (Wilson Hardness; ITW Test and Measurement GmbH, Shanghai, China) was used. A pyramid-shaped diamond indenter with a 50 g load and a dwell time of 30 seconds was used to create indents on three different areas of each specimen. The average value of the indentation hardness was calculated and utilised for subsequent analysis.

Statistical Package for Social Sciences (SPSS v. 23, IBM, USA) was used for all data analyses. Distribution of surface roughness values was not normal, therefore Kruskal-Wallis test was used for analysis, while one-way analysis of variance (ANOVA) with Tukey’s post-hoc test were used for hardness values. Significance was set at *p* < 0.05.

## Results

### Hardness

FormLabs and NextDent specimens’ hardness values indicate that both were significantly influenced by the type of printer used (*p* < 0.001), while AISGA specimens had similar hardness when printed with ASIGA or NextDent printer (*p* = 0.072). Hardness values of FormLabs and NextDent specimens improved when printed with ASIGA printer, compared to those printed with NextDent.

Printing with ASIGA produced similar hardness values for ASIGA and NextDent, which were higher than FormLabs specimens. Printing with NextDent showed the highest hardness value for AISGA specimens, with no significant difference between FormLabs and NextDent. Detailed hardness mean values and statistical interactions are summarised in Table 3.

**Table 3:** Mean (SD) and significances of hardness values of the tested groups.

Printer	ASIGA	FormLabs	NextDent	*P*
ASIGA	27.45 (2.59)^a^	24.19 (2.39)^b^	27.2 (3.43)^a^	< 0.001*
NextDent	29.06 (2.9)^a^	19.37 (2.65)^b^	20.8 (2.31)^b^	< 0.001*
*P*	0.072	< 0.001*	< 0.001*	

* Statistically significant at 0.05 level of significance.

- Rows: Dissimilar letters indicate significant differences.

- Columns: Difference between two groups is indicated by the *p*-value.

Two-way ANOVA (Table 4) showed significant interactions among groups for the resin material and printer model, as well as the combined effect of both factors.

**Table 4:** Two-Way ANOVA results for hardness values.

Source	Type III sum of squares	Df	Mean square	*F*	*P*
Corrected Model	1534.560	5	306.912	40.951	0.000*
Intercept	73084.884	1	73084.884	9751.625	0.000*
Printer model	307.8083	1	307.8083	41.0705	0.000*
Resin material	866.5228	2	433.2614	57.8095	0.000*
Printer model * resin material	360.2288	2	180.1144	24.0324	0.000*
Error	854.3886	114	7.4946		
Total	75473.833	120			
Corrected Total	2388.949	119			

* Statistically significant at 0.05 level of significance.

### Surface roughness

Similar to hardness values, surface roughness measurements can be influenced by the type of 3D printer used. ASIGA and FormLabs specimens had higher surface roughness values when printed with ASIGA printer compared to when NextDent printer was used (*p* < 0.05). NextDent specimens, however, were not influenced by the type of printer used (*p* = 0.053).

When the same printer was used, FormLabs specimens showed the highest surface roughness values, and the lowest values were observed with ASIGA specimens (*P* < 0.001). All results are summarised in Table 5.

**Table 5:** Mean (SD) and significances of roughness values of the tested groups.

Printer	ASIGA	FormLabs	NextDent	*P*
ASIGA printer	0.48 (0.07)^a^	0.73 (0.04)^b^	0.57 (0.09)^c^	< 0.001*
NextDent printer	0.42 (0.07)^a^	0.63 (0.1)^b^	0.51 (0.1)^c^	< 0.001*
*P*	0.010*	< 0.001*	0.053	

* Statistically significant at 0.05 level of significance.

- Rows: Dissimilar letters indicate significant differences.

- Columns: Difference between two groups is indicated by the *p*-value.

Two-way ANOVA (Table 6) showed significant interactions for the type of printer and the resin material used; however, the combined effect of both factors was not significant (*p* = 0.387).

**Table 6:** Two-Way ANOVA results for surface roughness values.

Source	Type III sum of squares	Df	Mean square	*F*	*P*
Corrected Model	1.255	5	0.251	39.323	0.000^*^
Intercept	36.919	1	36.919	5784.118	0.000^*^
Printer model	0.164	1	0.164	25.669	0.000^*^
Resin material	1.079	2	0.539	84.515	0.000^*^
Printer model * resin material	0.012	2	0.006	0.957	0.387
Error	0.728	114	0.006		
Total	38.901	120			
Corrected Total	1.983	119			

* Statistically significant at 0.05 level of significance.

## Discussion

The findings of this investigation reveal significant variations in microhardness and surface roughness of 3D-printed denture base resins, attributable to both the resin material and the specific 3D printing system used. Therefore, the null hypothesis has been rejected. The observed differences underscore the critical importance of carefully selecting both the resin material and the printing technology to achieve optimal mechanical and surface characteristics for clinical applications.

Microhardness of denture base resins influences the material resistance to permanent deformation, which affects denture treatment longevity [30]. In this study, using different 3D printers had no influence on the microhardness of ASIGA specimens, which had similar values regardless of the printing system. FormLabs specimens, however, exhibited significantly higher hardness values when printed with ASIGA printer compared to NextDent printer. Interestingly, the same observation was noted with NextDent specimens, which demonstrated superior microhardness when printed with ASIGA printer compared to the same-brand and manufacturer-recommended printer. This may be attributed to the longer post-curing time recommended for ASIGA printers, compared to those recommended for NextDent printers. This has been confirmed in a previous study also where longer post-curing time produced higher microhardness values [29]. Another factor is the distinct polymerisation kinetics inherent to each printing system, potentially leading to different cross-linked polymer networks. Previous investigations have indicated that printer type significantly affects mechanical properties, including flexural strength, flexural modulus, and microhardness [31, 32]. This suggests that the choice of printer and resin material combination critically impacts the material’s structural integrity and long-term clinical performance.

Surface roughness of denture base can influence colour stability, bacterial adhesion, and biofilm formation, which are critical factors for treatment success and proper hygiene of dental prostheses [33, 34]. The results of this study indicate that ASIGA specimens consistently demonstrated the lowest surface roughness values, regardless of the printer utilised, aligning with previous research that highlights ASIGA’s superior surface characteristics among 3D-printed resins [35, 36]. The use of different printers influenced resultant surface roughness values of ASIGA and FormLabs specimens, unlike NextDent specimens, which were not affected by the type of printer used. The use of NextDent printer produced superior surface roughness values for both FormLabs and ASIGA specimens, compared to ASIGA printers. The use of ASIGA printers has been reported to produce higher surface roughness of 3D printed resin used for occlusal guards [32], compared to Liquid Crystal Display (LCD) printer. The study reported that the difference may be due to different light intensities of the two printing systems. However, another *in vitro* study reported comparable surface roughness of denture base specimens when printed with either DLP or LCD printer [28]. In the present study, both printing systems use DLP technology, which showed different surface roughness in only two of the three tested resin materials. Therefore, the choice of 3D printed resin and the printing system can influence surface roughness of denture base resins. The conflict in results indicates that other factors beyond printer and resin, such as printing parameters and post-curing process, may have an impact on the surface characteristics of denture base resins, which warrants further investigations.

The results of the current study showed that using the manufacturer-recommended printer does not necessarily produce superior properties when compared to third-party printer. Microhardness values for NextDent specimens were higher when printed with the ASIGA system, compared to those printed with their own NextDent system. Similarly, surface roughness values for ASIGA specimens were superior when printed with NextDent printing system, compared to when ASIGA printer was used. In contrast, a previous study [27] compared same-brand printer with third-party printer and reported that using the manufacturer-recommended 3D printer exhibited significantly higher mechanical properties, including ultimate strength, elastic modulus, and hardness than those printed with third-party 3D-printer. In addition, resin specimens printed with the recommended 3D-printer had significantly smoother surfaces than those printed with a third-party 3D-printer. It is worth mentioning that the manufacturer-recommended printer was DLP system, while the third-party printer utilised an SLA technology. Thus, the observed differences may be attributable to differences in printing technologies rather than the resin-printer combination. Therefore, a comprehensive understanding of printer technologies and their interaction with different resin materials, beyond merely adherence to manufacturer recommendations, is crucial for optimising the final material properties. It is important to note that while some differences in microhardness and surface roughness values in the present study reached statistical significance, their clinical relevance remains undetermined.

Study limitations include the fact that the results are true only for the materials/printers tested. Factors simulating complex oral environment, such as thermal and pH cycling were not incorporated and warrants further investigations. To gain a comprehensive understanding of how different printers/technologies impact material properties, evaluating degree of conversion, printing parameters and post curing time is essential. Further investigations are needed to evaluate various combinations of resin materials, printers, and printing technologies, which may be a step towards improving the inferior physical and mechanical properties of 3D printed resins compared to milled ones.

## Conclusions

Within the limitations of the study, the following can be concluded:

1. The choice of the resin material and 3D printer significantly impacts the microhardness and surface roughness of 3D-printed denture base resins.
2. Using a manufacturer’s recommended printer does not guarantee superior mechanical properties or surface characteristics.
3. The interactions between specific resin material and printer types seem complex. Further studies are needed to reach a conclusive understanding.

## Data Availability

The data supporting this study’s findings are available from the corresponding author upon reasonable request.
